# *Severe Acute Respiratory Syndrome Coronavirus 2* (SARS-CoV-2) Exhibits High Predicted Binding Affinity to ACE2 from Lagomorphs (Rabbits and Pikas)

**DOI:** 10.3390/ani10091460

**Published:** 2020-08-20

**Authors:** Silvia Preziuso

**Affiliations:** School of Biosciences and Veterinary Medicine, University of Camerino, 62032 Camerino, Italy; silvia.preziuso@unicam.it

**Keywords:** SARS-CoV-2, ACE2, lagomorphs, virus–receptor complex, *in silico*

## Abstract

**Simple Summary:**

*Severe acute respiratory syndrome coronavirus 2* (SARS-CoV-2) is responsible for the pandemic COVID-19. The virus infects human cells by binding of the virus spike to the cell receptor ACE2. Some studies suggest that dogs, cats and other animal species could be infected by SARS-CoV-2, while very limited data are available on lagomorphs. There are several occasions where rabbits and other lagomorphs are in close contact with humans. To investigate the interaction between SARS-CoV-2 spikes and ACE2 of lagomorphs, predictive computer-based models were used in this study. The structure of ACE2 of lagomorphs was obtained on the basis of the amino acid sequences computationally. The interaction with the model of SARS-CoV-2 spikes was studied and described in depth on the basis of the complex human ACE2-SARS-CoV-2 published before. The interaction among SARS-CoV-2 spikes and ACE2 of other companion or laboratory animals is also described for comparative purposes. The results predict that ACE2 of lagomorphs are likely to bind SARS-CoV-2 spikes and suggest that further studies would be justified to confirm these results and to evaluate the risks to humans being in close contact with lagomorphs, such as veterinarians, farmers, slaughterhouse workers, butchers or pet owners.

**Abstract:**

*Severe acute respiratory syndrome coronavirus 2* (SARS-CoV-2) is responsible for the pandemic COVID-19. The virus infects human cells by binding of the virus spike to the cell receptor ACE2. The crystal structure of SARS-CoV-2 spikes in complex with human ACE2 has recently been solved, and the main amino acid residues involved in the virus–receptor complex have been detected. To investigate the affinity of ACE2 of lagomorphs to the SARS-CoV-2 spike, ACE2 sequences from rabbits and American pikas were compared with human ACE2 and with ACE2 from mammals with different susceptibility to the virus. Models of the complex formed by SARS-CoV-2 spike and ACE2 from lagomorphs and from other mammals were created for comparative studies. ACE2 of lagomorphs showed fewer substitutions than human ACE2 in residues involved in the ACE2-SARS-CoV-2 spike complex, similar to cats. Analysis of the binding interface of the simulated complexes ACE2-SARS-CoV-2 spike showed high affinity of the ACE2 of lagomorphs to the viral spike protein. These findings suggest that the spike of SARS-CoV-2 could bind the ACE2 receptor of lagomorphs, and future studies should investigate the role of lagomorphs in SARS-CoV-2 epidemiology. Furthermore, the risks to humans coming into close contacts with these animals should be evaluated.

## 1. Introduction

Since its discovery in China, *Severe acute respiratory syndrome coronavirus 2* (SARS-CoV-2) has been spreading rapidly worldwide, causing the Coronavirus Disease 2019 (COVID-19) pandemic [1]. SARS-CoV-2 belongs to the genus *Betacoronavirus* and shows very high sequence similarity to bat *Rhinolophus affinis* coronavirus RaTG13 in the whole genome (93.7% amino acid similarity) [2] and to the Guangdong pangolin coronaviruses in the receptor-binding domain (RBD) [3,4]. RBD is part of the C-terminal domain of the S1 subunit in the SARS-CoV-2 spike protein (S), which interacts with the human angiotensin-converting enzyme 2 (hACE2) receptor [5,6]. The structure of SARS-CoV-2 spike has been investigated by cryo-electron microscopy, and high affinity for hACE2 was found [7]. Priming of coronavirus spike proteins by host cell proteases is essential for viral entry into cells. For example, the cellular serine protease TMPRSS2 is employed by SARS-CoV-2 for the priming of the spike protein [8]. Host protease processing acts as a species barrier, and the addition of exogenous protease to cell cultures may facilitate coronaviruses cell entry. However, the viral spike does not enter the cells, regardless of protease addition, if the receptor is absent or incompatible [9]. Despite other entry mechanisms and potential receptors are under investigations [10,11], the angiotensin-converting enzyme 2 (ACE2) is considered the main receptor for SARS-CoV2 so far. ACE2 protein is abundantly expressed not only in different cells of the human respiratory tract, but also in many other cells in several organs [12]. hACE2 is also the receptor of the other *Betacoronavirus* SARS-CoV, which caused the severe acute respiratory syndrome (SARS) epidemic in 26 countries in 2002–2003 [13].

The crystal structure of SARS-CoV-2 RBD in complex with its receptor hACE2 has been solved (PBD ID 6LZG, PDB 6M17) [6,14]. Despite the overall similarity between the complexes SARS-CoV-RBD and SARS-CoV-2-RBD, several sequence variations and conformational deviations were found in their respective interfaces with ACE2 [14]. Furthermore, the SARS-CoV-2-RBD binding interface has more residues than SARS-RBD (21 versus 17) that directly interact with hACE2, forming more Van der Waals contacts and H-bonds and consequently forming a stronger binding [6]. The specific binding with its receptor determines the host range of a virus. Investigating animal species susceptibility to SARS-CoV-2 is helpful for evaluating the risks of transmission between humans and animals and to prevent further spread of the epidemic, although entry and post-entry barriers may limit the virus replication. Studies based on experimental infections in animals require high-level biosafety facilities and pose ethical issues and legislative concerns. Preliminary screening of potential susceptible animal species is useful before planning in vitro and in vivo experiments. The animal host range of SARS-CoV-2 has been predicted by sequence analysis and structure simulation studies [15,16]; however, not all key residues at the SARS-CoV-2-ACE2 interface, but rather only a limited number of them, have been considered in these studies. Evidence of SARS-CoV-2 infection in animals was reviewed while this article was under revision [17,18]. Lagomorphs may act as an important wildlife reservoir for zoonotic viruses. In vitro and in vivo studies on interaction between SARS-CoV-2 and ACE2 of lagomorphs are lacking, despite the common use of these animals in laboratories, as pets, for food production or for hunting.

In this study, the ACE2 sequences of lagomorphs were investigated and the binding interfaces of the complexes SARS-CoV-2-ACE2 of lagomorphs were predicted in comparison with humans. In addition, comparison was carried out with SARS-CoV-2-ACE2 models of some mammals which showed different degrees of susceptibility to the virus in recent studies.

## 2. Materials and Methods

### 2.1. Sequence Analysis

ACE2 sequences from lagomorphs available in databases (rabbit *Oryctolagus cuniculus* XP_002719891 and American pika *Ochotona princeps* XP_004597549.2) were downloaded. These sequences were compared with ACE2 sequences from other companion mammals showing different degrees of susceptibility to SARS-CoV-2 as dogs (*Canis lupus familiaris* NP_001158732.1), cats (*Felis catus* XP_023104564.1), ferrets (*Mustela putorius furo* NP_001297119.1), Chinese hamsters (*Cricetulus griseus* XP_003503283.1), golden Syrian hamsters (*Mesocricetus auratus* XP_005074266.1) [16,17,18,19,20,21,22,23]. Sequences of mice (*Mus musculus* NP_081562.2) and brown rats (*Rattus norvegicus* NP_001012006.1) were also considered because recent structural studies suggest that mouse and rat ACE2 are likely poor receptors for SARS-CoV-2 [5,16]. Sequence of the human ACE2 was used as reference sequence (NP_001358344.1).

Pairwise alignment of amino acid sequences was achieved using computer program MUSCLE (https://www.ebi.ac.uk/Tools/msa/muscle/). Sequences were edited with BioEdit software version 7.2 [24]. ACE2 phylogenetic tree was inferred with the software MEGA version 10.1 [25]. The estimated best-fitting substitution model was Jones-Taylor-Thornton with gamma-distributed rates among sites. Then the maximum likelihood method with bootstrap values based on 1000 repetitions was used. Pairwise distances between the sequences were calculated with the software MEGA version 10.1.

### 2.2. Structure Simulation of the Complex SARS-CoV-2-RBD-ACE2

The 3D structure of the SARS-CoV-2-RBD-ACE2 complex for each animal species was simulated by homology modelling using SWISS-MODEL [26]. The structure of SARS-CoV-2 spike RBD complexed with its receptor ACE2 was used as template (PDB ID: 6LZG) [6]. The obtained structures were analyzed by Chimera software version 1.14 [27].

Prediction of binding affinity and different types of intermolecular interactions was carried out with the web server PRODIGY (PROtein binDIng enerGY prediction) (https://bianca.science.uu.nl/prodigy/) [28,29]. This web application predicts the binding affinity (BA, expressed as Gibbs free energy ΔG; the lower the value is, the stronger the predicted BA is), counts the number of interfacial contacts (ICs) made at the interface of a protein–protein complex within a 5.5 Å distance threshold, and classifies them according to the polar/nonpolar/charged character of the interacting amino acids. This information is then combined with properties on the Non-Interacting Surface (NIS), which can influence the BA [28,30].

## 3. Results

### 3.1. Sequence Analysis

**Figure 1:**
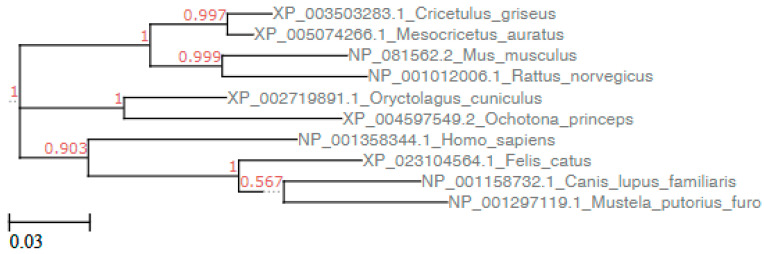
Phylogenetic analysis of the ACE2 sequences of lagomorphs and of other mammals. Human ACE2 sequence was used as reference sequence. The phylogenetic analysis was performed with a maximum likelihood (ML) method using the Jones-Taylor-Thornton model with a gamma distribution and with bootstrap values based on 1000. The tree scale is reported on the bottom left. The tree was visualized by the online tool ETE Toolkit (www.etetoolkit.org).

Amino acid sequence alignment of ACE2 molecules from humans, dogs, cats, rabbits, American pikas, Chinese hamsters, golden Syrian hamsters, mice, brown rat, and ferret showed changes in many residues all along the 807 aa sequences. Phylogenetic and pair distance analysis showed that whole ACE2 sequences of rabbits and cats were respectively the first and the second sequences more related with hACE2, while American pikas ACE2 was the most dissimilar to hACE2 (Figure 1 and Table 1). However, not all ACE2 amino acids are involved in the complex with SARS-CoV-2-RBD, being only 22 residues involved in the binding with SARS-CoV-2-RBD [6]. The positions of the key residues involved in the binding with SARS-CoV-2-RBD are reported in the first row of Table 2. A few changes were found among these 22 ACE2 residues in different animal species (Table 2). When only these residues were considered, Chinese hamster and golden Syrian hamster ACE2 were the closest to hACE2 sequence, followed by cats, rabbits and pikas, while mouse and rat ACE2 were the most dissimilar to hACE2 (Table 2, Supplementary Table S1). Among the 22 residues involved in the binding with SARS-CoV-2-RBD [6], ACE2 of Chinese hamsters and golden Syrian hamsters showed only 2 changes (H34Q and M82N). ACE2 of rabbits, American pikas, cats and dogs showed 4 changes, ACE2 of ferrets showed seven changes, and ACE2 of mice and rats showed eight changes. Most changes among sequences from different animal species were found at ACE residues Q24, D30 H34 and M82 (Table 2). The effect of a change in the sequence can be favorable, unfavorable or neutral. The consequences of changes in key residues of the ACE2 sequences were predicted by structure simulations.

### 3.2. Structure Simulation of the Complex SARS-CoV-2-RBD-ACE2

**Figure 2:**
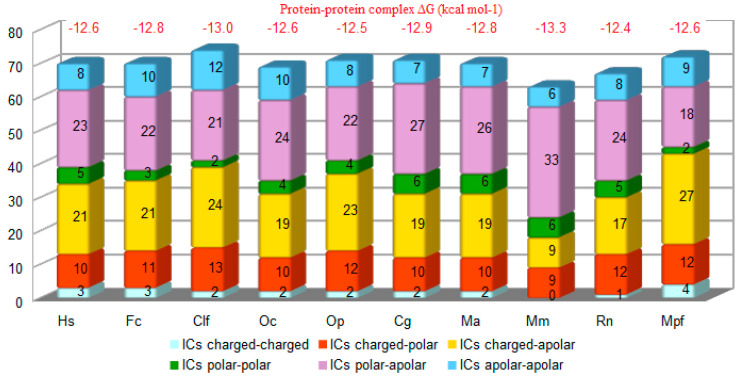
Interfacial contacts (ICs) (axis Y) observed in the ACE2-SARS-CoV-2 predicted complex in humans (Hs), cats (Fc), dogs (Clf), rabbit (Oc), pika (Op), Chinese hamster (Cg), golden Syrian hamster (Ma), ferrets (Mpf), mouse (Mm), rat (Rn) (axis X) by PRODIGY. Predicted protein–protein complex binding affinity expressed as Gibbs free energy (ΔG; kcal mol-1) for each complex is reported in red on the top of each column.

**Figure 3:**
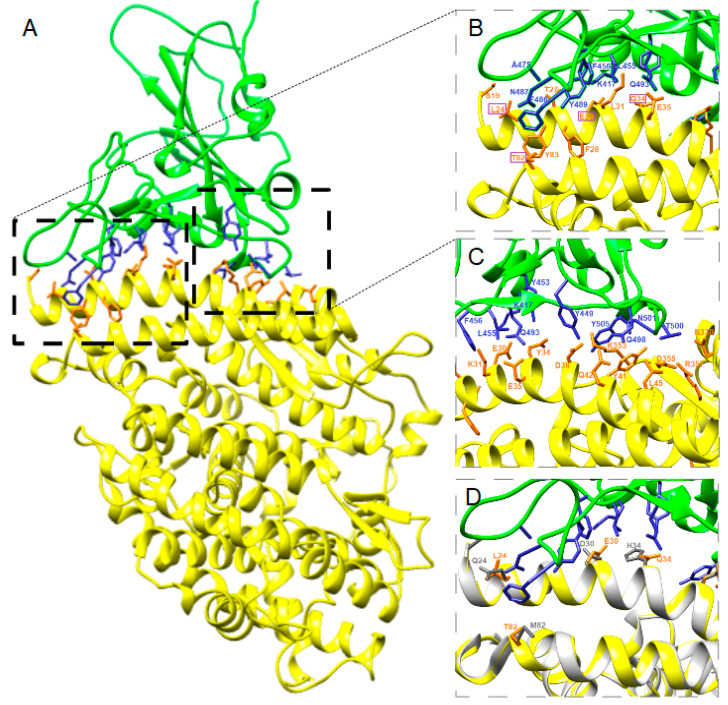
Predicted complex (**A**) between rabbit ACE2 (yellow) and spike SARS-CoV-2-RBD (green). The main amino acid interactions included in black rectangles are further reported in (**B**) and (**C**). Spike SARS-CoV-2-RBD residues are colored blue and ACE2 residues are colored orange. Purple rectangles highlight changed amino acids in comparison with the hACE2 sequence. Differences among hACE and rabbit ACE2 residues involved in the complex with SARS-CoV-2-RBD are shown in (**D**). hACE is colored white and its residues are colored grey, rabbit ACE2 is colored yellow and its residues are colored orange, SARS-CoV-2-RBD is colored green and its residues are colored blue.

**Figure 4:**
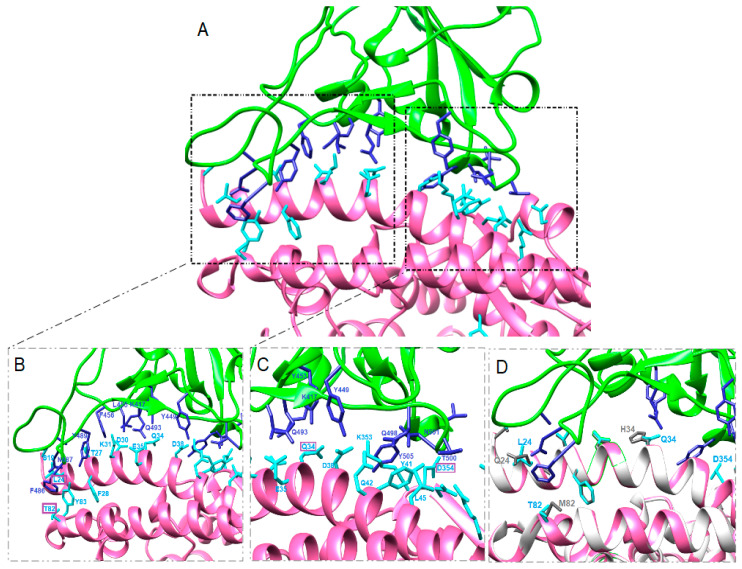
Predicted complex (**A**) between American pika ACE2 (pink) and SARS-CoV-2-RBD (green). The main amino acid interactions included in black rectangles are further reported in (**B**) and (**C**). SARS-CoV-2 residues are colored blue and ACE2 residues are colored cyan. Purple rectangles highlight changed amino acids in comparison with the hACE2 sequence. Differences among hACE and American pika ACE2 residues involved in the complex with SARS-CoV-2-RBD are shown in (**D**). hACE is colored white and its residues are colored grey, American pika ACE2 is colored pink and its residues are colored cyan, SARS-CoV-2 is colored green and its residues are colored blue.

The structure of the protein complex between SARS-CoV-2-RBD and ACE2 of cats, dogs, rabbits, American pikas, Chinese hamsters, golden Syrian hamsters, mice, rats and ferrets were predicted by using the whole sequence of ACE2 (Figure 2, Figure 3 and Figure 4 and Figure S1).

The positions of the ACE2 residues involved in the complex with SARS-CoV-2-RBD in animals were the same as the ACE2 residues involved in the complex with SARS-CoV-2-RBD in humans (listed in the first column of Table S2). The number of contacts predicted by both Chimera and PRODIGY between ACE2 and SARS-CoV-2-RBD was higher in all animals, apart from mice and rats, than in humans (Figure 2). In particular, the total number of the three strongest types of contacts charged-charged, charged-polar and charged-nonpolar in animal ACE2 complexes with spike SARS-CoV-2-RBD was similar to humans except for mice, which showed the lowest number (*n* = 18), and ferrets, which showed the highest number (*n* = 43). Thus, the number of contacts predicted between SARS-CoV-2-RBD and ACE2 of animal species proved to be more susceptible to the virus infection was higher than in animals proved to be less susceptible to the virus infection. This difference is evident in particular when only the three strongest type of contacts are considered. Predicted interactions between ACE2 and spike SARS-CoV-2-RBD showed that BAs expressed as Gibbs free energy values in rabbits and American pikas were similar to humans (Figure 2).

All the animal species included in the study showed similar BAs calculated by the PRODIGY method, suggesting that the spike protein of SARS-CoV-2 could have similar affinity for ACE2 of mammals. However, the lowest BA value was obtained in mice, which is considered a species with low susceptibility to SARS-CoV-2 infection [31]. Further investigations are required to evaluate whether the BAs values obtained by the PRODIGY method could be useful for predicting the interaction between spike SARS-CoV-2-RBD and ACE2 of different animals species.

The predicted key residues involved in ACE2-SARS-CoV-2-RBD complex formation in animals and the predicted distances are reported in the Supplementary Table S2. In our models, Q24 hACE was changed in all but Chinese and golden Syrian hamster ACE2 and all Q24L, Q24N and Q24K changes corresponded to a longer predicted distance with viral N487 (Table 2 and Supplementary Table S2). Similarly, D30N changes corresponded to lack of interaction with viral K417 in mice and rats, and in general D30E changes corresponded to a longer distance with viral K417 in cats, dogs and ferrets, but a shorter distance in rabbits. Shorter distance between ACE2 and spike SARS-CoV-2-RBD residues was found when H34Q substitution was present, while the change H34Y detected in dogs and ferrets corresponded to a longer distance. A longer distance was found also between Y449 and the changed residue D38E in cats, dogs and ferrets. Residue M82 was changed in all animals examined. The distance between viral F486 and T82 was shorter than M82, while the distance between viral F486 and N82 or S82 was longer than M82 (Supplementary Table S2).

The predicted structures of the protein complexes ACE2-SARS-CoV-2-RBD in rabbits and pikas showed that the four changes present in the respective ACE2 sequences do not affect significantly the binding between amino acids in ACE2 and in SARS-CoV-2-RBD in comparison with humans (Figure 3 and Figure 4). Indeed, some contacts between SARS-CoV-2-RBD and ACE2 of rabbits or American pikas were closer than those observed between SARS-CoV-2-RBD and hACE2, suggesting that changes in the ACE2 sequences could result in closer binding. Sequence studies should not be limited at detecting changes in the ACE2 sequences, but the consequences of these changes on the virus-receptor complex should be evaluated.

## 4. Discussion

SARS-CoV-2 is responsible for a pandemic and, in the absence of a vaccine, the only measures for prevention of COVID-19 mostly consist of avoiding direct and indirect contact between infected and susceptible humans. SARS-CoV-2 originated from bats, and pangolins have been proposed as intermediate hosts [3,4]. To assess the susceptibility of different animal species to SARS-CoV-2 is important not only for epidemiological purposes, but also for risk evaluation and management. Different animals are kept as pets and live in close contact with humans. Since from the first cases of COVID-19, pet owners have been asking scientists whether animals can spread the virus to people. The risk of human-to-animal transmission of SARS-CoV-2 and then the risk of animal-to-human transmission need to be investigated. In vivo studies by experimental infection of candidate animal hosts have many limitations due to ethical, legal and safety issues. Furthermore, experimental infections to assess the susceptibility of animals to SARS-CoV-2 are limited by the limited availability of high-level biosafety facilities and trained workers. Predictive models are helpful to identify the animal species that are more likely infectable by the virus. Furthermore, interpretation of data obtained by predictive models together with results obtained by in vitro and in vivo studies can suggest the key amino acid residues that play a role in the virus–receptor interaction. In this study, the most recent knowledge about the complex SARS-CoV-2-RBD-hACE2 [6] was used to predict the susceptibility of lagomorph animals in comparison with other mammals showing different degree of susceptibility to the virus by *in silico*, in vitro or in vivo investigations [16,17,18,19,20,21,22,23,32].

The complex structure between spike SARS-CoV-2-RBD and hACE2 includes strong polar contacts among key residues [6]. SARS-CoV-2-RBD residue A475 interacts with hACE2 residue S19, N487 with Q24, E484 with K31, and Y453 with H34. Residue K417 contributes ionic interactions with hACE2 D30. Several residues (G446, Y449, G496, Q498, T500, and G502) in the viral bulged loops are in close proximity with hACE2 amino acids D38, Y41, Q42, K353, and D355, forming a concentration of H-bonds. Further virus–receptor contacts include SARS-CoV-2-RBD Y489 and F486 packing against hACE2 residues F28, L79, M82, and Y83, forming a small patch of hydrophobic interactions at the interface [6]. Changes in these residues could interfere with the optimal binding between viral RBD and ACE2.

Despite the genetic variability of animal ACE2 sequences, the spike SARS-CoV-2-RBD is predicted to bind very conserved residues among animals. A lower number of changes in the ACE2 residues involved in the receptor-virus complex was detected in Chinese hamsters and in golden Syrian hamsters in comparison with humans, as reported recently [32]. The only two changes were M82N, corresponding to a longer distance with the viral residue F486, and H34Q, corresponding to a closer contact with the viral amino acid Y453 (Supplementary Table S2). Recent experiments have shown that golden Syrian hamsters are susceptible to SARS-CoV-2 infection, and they developed clinical signs and lesions and infected naïve contact hamsters housed in the same cage in a 1:1 ratio with infected hamsters [19]. High viral titer was found in the lungs, and the virus was also found in the intestine. These findings suggest that SARS-CoV-2 infection in golden Syrian hamsters occurs despite the M82N and H34Q changes.

American pikas showed two changes at the same positions as golden Syrian hamsters in ACE2. In addition to the same H34Q substitution of golden Syrian hamsters, American pikas have a change at M82T, which shows closer contact with F486 than humans and hamsters (Supplementary Table S2). The same M82T substitution is also present in cats, dogs and ferrets, but not in rats and mice. American pika ACE2 also has the favorable change G354D, and thus a total of three out of four changes result in closer contacts with their viral residues. The fourth substitution in American pika ACE2 is Q24L, which together with M82T is present also in cats, dogs and ferrets, but not in rats and mice.

Recent SARS-CoV-2 experimental infections in cats showed that the virus can replicate efficiently, younger cats are more permissive, and airborne viral transmission between cats may occur [20]. Cats and rabbits share three out of four changes in the SARS-CoV-2 spike-contacting regions of ACE2. The fourth change in rabbit is H34Q, which corresponds to a shorter distance between Q34 and Y453 in all animals having this substitution. In comparison, the fourth change in cats is D38E, corresponding to a longer distance between E38 and the viral amino acid Y449. Furthermore, the predicted protein–protein complex ΔG in rabbits is the same as in humans. These findings suggest that SARS-CoV-2-RBD could bind ACE2 receptor of rabbits and American pikas similarly to in humans, although these findings need to be confirmed.

Experimental studies also showed that SARS-CoV-2 can replicate in the upper respiratory tract of ferrets for up to eight days, but not in the lungs or in other organs. Dogs were found to have low susceptibility to SARS-CoV-2 because the virus was found only sporadically in rectal swabs and not in the respiratory tract [20]. Dogs and cats have the same amino acid differences in the SARS-CoV-2 spike-contacting regions of ACE2, but dogs have the extra change H34Y, which is absent in cats. This substitution corresponds to a longer distance between Y34 and viral Y453 and could interfere with efficient virus binding. H34Y change is also present in ferrets, which showed less efficient viral replication than cats [20].

As observed about SARS-CoV [33] and SARS-CoV-2 [31], mice and rats are not ideal model animals for studying SARS-CoV-2. Although based on the analysis of a small number of residues, some studies have suggested that mice and efficiently rats could not bind SARS-CoV-2 RBD [5,15,16]. In particular, mouse or rat ACE2 contains an asparagine at the 30 position and a histidine at the 353 position, which does not fit into the virus–receptor interaction as well as aspartate or a lysine do, respectively. Furthermore, three changes are present at positions L79, M82 and Y83, which together with F28 form a small patch of hydrophobic interactions at the interface. These findings, in addition to the low number of the interfacial contacts and the number of charged-charged, charged-polar and charged-nonpolar contacts found in this study, support the hypothesis that ACE2 of mice and rats are not optimal receptors for SARS-CoV-2. Experimental infection of wild type mice and of hACE2 transgenic mice confirmed that human ACE2 is required to establish an effective infection [31].

## 5. Conclusions

In conclusion, the simulated complexes ACE2-SARS-CoV-2-RBD showed high affinity of ACE2 of lagomorphs to the viral spike protein, suggesting that lagomorphs could be susceptible to SARS-CoV-2. There are several occasions where rabbits can be in close contact with humans. For example, rabbits are reared and used for food in many countries, and thus farmers, slaughterhouse workers, veterinarians, butchers, etc. could be exposed to the virus. Furthermore, different species of rabbits are kept as pet animals and the risk for veterinarians should be evaluated when they examinate rabbits living with symptomatic or asymptomatic SARS-CoV-2-infected rabbit owners. The recent finding of the potential for pangolins as the zoonotic reservoir of SARS-CoV-2-like coronaviruses raises the question of the role of wildlife in the epidemiology of the virus. Pikas distribution varies greatly based on species. Two species live in North America, the rest range throughout Asia. Pikas are wild animals, they are not usually kept as pets but are housed in zoos. Some pika species are at risk of extinction due to attempts to capture them and to breed them in captivity. Other lagomorphs are wild animals. Unfortunately, only two sequences of ACE2 of lagomorphs (*Oryctolagus cuniculus* and *Ochotona princeps*) are available in GenBank so far. Future studies should be aimed at sequencing ACE2 protein from different species of lagomorphs. In silico predictions are helpful to detect the animal species having receptors with highest affinity for the virus and to predict favorable and unfavorable virus–receptor interactions. Predicted positive interactions should be confirmed by in vitro and in vivo studies because replication of viruses in species other than their natural hosts can be limited by entry and post-entry barriers.

## Figures and Tables

**Table 1:** Estimates of evolutionary divergence between sequences. The whole 807 amino acid long sequences of ACE2were considered. The number of amino acid substitutions per site between sequences are shown. Analyses were conducted using the JTT matrix-based model. The rate variation among sites was modeled with a gamma distribution (shape parameter = 5).

ACE2 Sequence		1	2	3	4	5	6	7	8	9
1	NP_001358344.1_Homo_sapiens	-								
2	XP_023104564.1_Felis_catus	0.170	-							
3	NP_001158732.1_Canis_lupus_familiaris	0.188	0.105	-						
4	XP_002719891.1_Oryctolagus_cuniculus	0.164	0.179	0.187	-					
5	XP_004597549.2_Ochotona_princeps	0.219	0.224	0.220	0.126	-				
6	XP_003503283.1_Cricetulus_griseus	0.174	0.195	0.198	0.149	0.178	-			
7	XP_005074266.1_Mesocricetus_auratus	0.172	0.200	0.204	0.147	0.175	0.029	-		
8	NP_081562.2_Mus_musculus	0.204	0.213	0.219	0.183	0.205	0.114	0.109	-	
9	NP_001012006.1_Rattus_norvegicus	0.201	0.219	0.229	0.183	0.210	0.118	0.112	0.103	-
10	NP_001297119.1_Mustela_putorius_furo	0.199	0.115	0.112	0.195	0.232	0.201	0.206	0.216	0.235

**Table 2:** Alignment of ACE2 sequences and comparison among residues involved in the contact with SARS-CoV-2 RBD. Points mean that the amino acid is identical to the corresponding amino acid in the hACE2 sequence.

ACE2	Amino Acid Position										
	19	24	27	28	30	31	34	35	37	38	41
*Homo sapiens*	S	Q	T	F	D	K	H	E	E	D	Y
*Felis catus*	.	L	.	.	E	.	.	.	.	E	.
*Canis lupus familiaris*	.	L	.	.	E	.	Y	.	.	E	.
*Oryctolagus cuniculus*	.	L	.	.	E	.	Q	.	.	.	.
*Ochotona princeps*	.	L	.	.	.	.	Q	.	.	.	.
*Cricetulus griseus*	.	.	.	.	.	.	Q	.	.	.	.
*Mesocricetus auratus*	.	.	.	.	.	.	Q	.	.	.	.
*Mus musculus*	.	N	.	.	N	N	Q	.	.	.	.
*Rattus norvegicus*	.	K	S	.	N	.	Q	.	.	.	.
*Mustela putorius furio*	.	L	.		E	.	Y	.	.	E	.
**ACE2**	**Amino Acid Position**										
	**42**	**45**	**79**	**82**	**83**	**330**	**353**	**354**	**355**	**357**	**393**
*Homo sapiens*	Q	L	L	M	Y	N	K	G	D	R	R
*Felis catus*	.	.	.	T	.	.	.	.	.	.	.
*Canis lupus familiaris*	.	.	.	T	.	.	.	.	.	.	.
*Oryctolagus cuniculus*	.	.	.	T	.	.	.	.	.	.	.
*Ochotona princeps*	.	.	.	T	.	.	.	D	.	.	.
*Cricetulus griseus*	.	.	.	N	.	.	.	.	.	.	.
*Mesocricetus auratus*	.	.	.	N	.	.	.	.	.	.	.
*Mus musculus*	.	.	T	S	F	.	H	.	.	.	.
*Rattus norvegicus*	.	.	I	N	F	.	H	.	.	.	.
*Mustela putorius furio*	.	.	H	T	.	.	.	R	.	.	.

## Supplementary Materials

The following are available online at https://www.mdpi.com/2076-2615/10/9/1460/s1, Table S1: Estimates of Evolutionary Divergence between Sequences. Only 22 residues corresponding to the sites of contact between SARS-CoV-2 RBD and ACE2 were considered. The number of amino acid substitutions per site from between sequences are shown. Analyses were conducted using the JTT matrix-based model. The rate variation among sites was modeled with a gamma distribution (shape parameter = 5); Table S2: Predicted distances among residues involved in contacts between ACE2 and SARS-CoV-2 calculated by Chimera software. Data are reported only for residues showing substitutions. The lowest distance is reported when an ACE2 residue shows more than one contact with the same SARS-CoV-2 residue; Figure S1: Structure simulation of the complex SARS CoV-2-ACE2 of cats (A), dogs (B), Chinese hamsters (C), golden Syrian hamsters (D), mice (E), rats (F) and ferrets (G).
