# Supplementary material for: Severe Acute Respiratory Syndrome Coronavirus 2 (SARS-CoV-2) Exhibits High Predicted Binding Affinity to ACE2 from Lagomorphs (Rabbits and Pikas)
Source: Animals (Basel). 2020 Aug 20;10(9):1460. doi: 10.3390/ani10091460 (PMC7552617; doi:10.3390/ani10091460)
Supplement: Supplementary file 1 [file animals-10-01460-s001.zip › Manuscript_v2_Supplementary fles/Supplementary Figure S1.pdf]

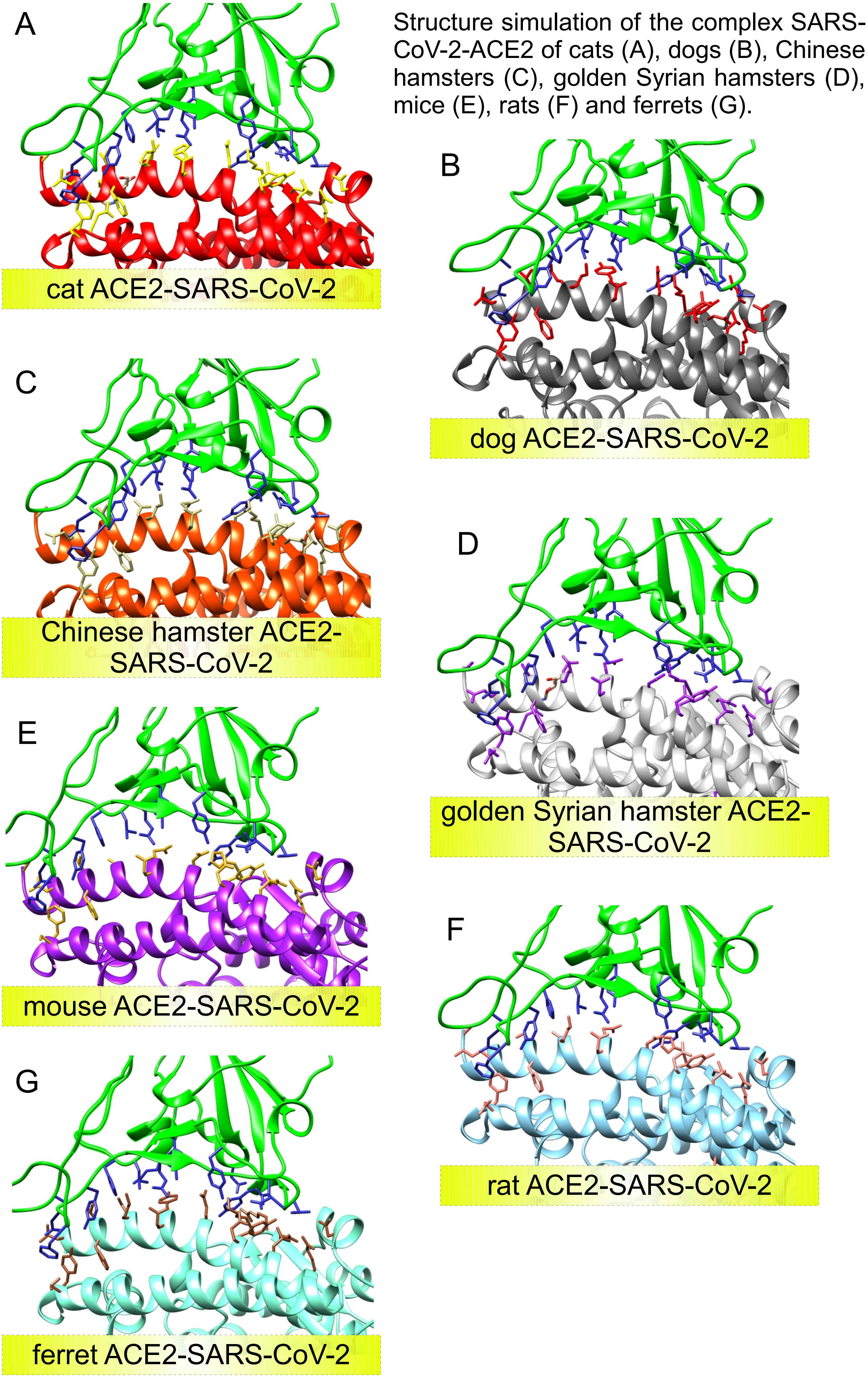

Structure simulation of the complex SARS-CoV-2-ACE2 of cats (A), dogs (B), Chinese hamsters (C), golden Syrian hamsters (D), mice (E), rats (F) and ferrets (G).
