# Supplementary material for: Severe Acute Respiratory Syndrome Coronavirus 2 (SARS-CoV-2) Exhibits High Predicted Binding Affinity to ACE2 from Lagomorphs (Rabbits and Pikas)
Source: Animals (Basel). 2020 Aug 20;10(9):1460. doi: 10.3390/ani10091460 (PMC7552617; doi:10.3390/ani10091460)
Supplement: Supplementary file 1 [file animals-10-01460-s001.zip › Manuscript_v2_Supplementary fles/Supplementary Table S1.pdf]

		1	2	3	4	5	6	7	8	9
**1**	NP_001358344.1_Homo_sapiens									
**2**	XP_023104564.1_Felis_catus	0.206								
**3**	NP_001158732.1_Canis_lupus_familiaris	0.265	0.049							
**4**	XP_002719891.1_Oryctolagus_cuniculus	0.207	0.098	0.109						
**5**	XP_004597549.2_Ochotona_princeps	0.209	0.204	0.224	0.099					
**6**	XP_003503283.1_Cricetulus_griseus	0.103	0.263	0.289	0.154	0.155				
**7**	XP_005074266.1_Mesocricetus_auratus	0.103	0.263	0.289	0.154	0.155	0			
**8**	NP_081562.2_Mus_musculus	0.549	0.639	0.695	0.494	0.53	0.433	0.433		
**9**	NP_001012006.1_Rattus_norvegicus	0.494	0.615	0.669	0.473	0.509	0.336	0.336	0.242	
**10**	NP_001297119.1_Mustela_putorius_furo	0.392	0.154	0.103	0.227	0.315	0.424	0.424	0.765	0.801

Estimates of Evolutionary Divergence between Sequences. Only 22 residues corresponding to the sites of contact between SARS-CoV-2 RBD and ACE2 were considered. The number of amino acid substitutions per site from between sequences are shown. Analyses were conducted using the JTT matrix-based model. The rate variation among sites was modeled with a gamma distribution (shape parameter = 5).
