# Supplementary material for: Severe Acute Respiratory Syndrome Coronavirus 2 (SARS-CoV-2) Exhibits High Predicted Binding Affinity to ACE2 from Lagomorphs (Rabbits and Pikas)
Source: Animals (Basel). 2020 Aug 20;10(9):1460. doi: 10.3390/ani10091460 (PMC7552617; doi:10.3390/ani10091460)
Supplement: Supplementary file 1 [file animals-10-01460-s001.zip › Manuscript_v2_Supplementary fles/Supplementary Table S2.pdf]

	Homo sapiens				*Felis catus*					*Canis lupus fam.*					*Oryctolagus cuniculus*				
**ACE2 residue**	**aa1**	**aa2**	**num**	**dist**	**aa1**	**aa2**	**num**	**dist**	**var**	**aa1**	**aa2**	**num**	**dist**	**var**	**aa1**	**aa2**	**num**	**dist**	**var**
**24**	GLN	ALA	475	3.695	LEU	ALA	475	3.079	-	LEU	ALA	475	3.073	-	LEU	ALA	475	3.060	-
	GLN	GLY	476	4.121	LEU	GLY	476	3.465	-	LEU	GLY	476	3.546	-	LEU	GLY	476	3.453	-
	GLN	ASN	487	2.954	LEU	ASN	487	3.617	+	LEU	ASN	487	3.745	+	LEU	ASN	487	3.610	+
**27**	THR	PHE	456	3.553															
	THR	ALA	475	3.890															
	THR	TYR	489	3.637															
**28**	PHE	TYR	489	3.526															
	PHE	TYR	489	3.531															
**30**	ASP	LYS	417	2.547	GLU	LYS	417	2.614	+	GLU	LYS	417	3.035	+	GLU	LYS	417	2.574	+
					GLU	LEU	455	3.328	-						GLU	LEU	455	3.302	-
	ASP	PHE	456	3.886	GLU	PHE	456	3.574	-	GLU	PHE	456	3.357	-	GLU	PHE	456	3.567	-
**31**	LYS	TYR	489	3.993															
**34**	HIS	TYR	453	2.936						TYR	TYR	453	3.055	+	GLN	TYR	453	2.944	=
	HIS	LEU	455	3.336						TYR	LEU	455	3.284	-	GLN	LEU	455	3.908	+
	HIS	GLN	493	3.606						TYR	GLN	493	3.707	+	GLN	GLN	493	3.694	+
**38**	ASP	TYR	449	2.560	GLU	TYR	449	3.339	+	GLU	TYR	449	3.344	+					
	ASP	GLY	496	3.357	GLU	GLY	496	2.592	-	GLU	GLY	496	2.600	-					
**41**	TYR	GLN	498	3.937															
	TYR	THR	500	2.866															
	TYR	ASN	501	2.831															
**42**	GLN	GLY	446	3.361															
	GLN	TYR	449	2.882															
	GLN	GLN	498	3.212															
**79**																			
**82**	MET	PHE	486	3.545	THR	PHE	486	2.968	-	THR	PHE	486	2.970	-	THR	PHE	486	2.976	-
**83**	TYR	PHE	486	3.232															
	TYR	ASN	487	2.947															
**353**	LYS	GLY	496	2.899															
	LYS	ASN	501	3.762															
	LYS	GLY	502	2.847															
	LYS	TYR	505	3.339															
**354**	GLY																		
	GLY																		
	GLY																		
	GLY																		
**355**	ASP	THR	500	3.503															
**357**	ARG	THR	500	3.437															

Distances among residues involved in contacts between ACE2 and SARS-CoV-2 calculated by Chimera software.
Data are reported only for residues showing substitutions.The lowest distance is reported when an ACE2 residue shows more than one contact with the same SARS-CoV-2 residue.

Legend: aa1 is ACE2 aminoacid; aa2 is SARS-CoV-2 RBD aminoacid; num is the number of the aa2 position in the SARS-CoV-2 spike protein; dist is the predicted distance between aa1 and aa2 expressed in Angstrom; var is the variation of the distance in comparison with humans

(+) means a longer distance, (-) means a shorter distance, (=) means that no significant difference between distances was observed in comparison with contacts between human ACE2 and SARS-CoV-2

	Homo sapiens				*Ochotona princeps*					*Cricetulus griseus*					*Mesocricetus auratus*				
**ACE2 residue**	**aa1**	**aa2**	**num**	**dist**	**aa1**	**aa2**	**num**	**dist**	**var**	**aa1**	**aa2**	**num**	**dist**	**var**	**aa1**	**aa2**	**num**	**dist**	**var**
**24**	GLN	ALA	475	3.695	LEU	ALA	475	3.073	-										
	GLN	GLY	476	4.121	LEU	GLY	476	3.459	-										
	GLN	ASN	487	2.954	LEU	ASN	487	3.624	+										
**27**	THR	PHE	456	3.553															
	THR	ALA	475	3.890															
	THR	TYR	489	3.637															
**28**	PHE	TYR	489	3.526															
	PHE	TYR	489	3.531															
**30**	ASP	LYS	417	2.547															
	ASP	PHE	456	3.886															
**31**	LYS	TYR	489	3.993															
**34**	HIS	TYR	453	2.936	GLN	TYR	453	2.914	-	GLN	TYR	453	2.925	-	GLN	TYR	453	2.925	-
	HIS	LEU	455	3.336	GLN	LEU	455	3.921	-	GLN	LEU	455	3.919	+	GLN	LEU	455	3.919	+
	HIS	GLN	493	3.606	GLN	GLN	493	3.694	+	GLN	GLN	493	3.691	+	GLN	GLN	493	3.691	+
**38**	ASP	TYR	449	2.560															
	ASP	GLY	496	3.357															
**41**	TYR	GLN	498	3.937															
	TYR	THR	500	2.866															
	TYR	ASN	501	2.831															
**42**	GLN	GLY	446	3.361															
	GLN	TYR	449	2.882															
	GLN	GLN	498	3.212															
**79**																			
**82**	MET	PHE	486	3.545	THR	PHE	486	2.960	-	ASN	PHE	486	3.575	+	ASN	PHE	486	3.575	+
**83**	TYR	PHE	486	3.232															
	TYR	ASN	487	2.947															
**353**	LYS	GLY	496	2.899															
	LYS	ASN	501	3.762															
	LYS	GLY	502	2.847															
	LYS	TYR	505	3.339															
**354**	GLY																		
	GLY				ASP	GLY	502	3.757	-										
	GLY				ASP	VAL	503	3.417	-										
	GLY				ASP	TYR	505	3.696	-										
**355**	ASP	THR	500	3.503															
**357**	ARG	THR	500	3.437															

	Homo sapiens				*Mus musculus*					*Rattus norvegicus*					*Mustela putorius furo*				
**ACE2 residue**	**aa1**	**aa2**	**num**	**dist**	**aa1**	**aa2**	**num**	**dist**	**var**	**aa1**	**aa2**	**num**	**dist**	**var**	**aa1**	**aa2**	**num**	**dist**	**var**
**24**	GLN	ALA	475	3.695	ASN	ALA	475	2.928	-						LEU	ALA	475	3.054	-
	GLN	GLY	476	4.121						LYS	GLY	476	3.840	+	LEU	GLY	476	3.518	-
	GLN	ASN	487	2.954	ASN	ASN	487	3.692	+	LYS	ASN	487	3.348	+	LEU	ASN	487	3.695	+
**27**	THR	PHE	456	3.553															
	THR	ALA	475	3.890						SER	ALA	475	3.376	-					
	THR	TYR	489	3.637															
**28**	PHE	TYR	489	3.526															
	PHE	TYR	489	3.531															
**30**	ASP	LYS	417	2.547											GLU	LYS	417	3.154	+
	ASP	PHE	456	3.886	ASN	PHE	456	3.850	-	ASN	PHE	456	3.857	-					
**31**	LYS	TYR	489	3.993	LYS	TYR	489	4.012	+										
**34**	HIS	TYR	453	2.936	GLN	TYR	453	2.876	-	GLN	TYR	453	2.886	-	TYR	TYR	453	3.054	+
	HIS	LEU	455	3.336	GLN	LEU	455	3.920	+	GLN	LEU	455	3.920	+	TYR	LEU	455	3.266	-
	HIS	GLN	493	3.606	GLN	GLN	493	3.684	+	GLN	GLN	493	3.697	+	TYR	GLN	493	3.649	+
**38**	ASP	TYR	449	2.560											GLU	TYR	449	3.353	+
	ASP	GLY	496	3.357											GLU	GLY	496	2.617	-
**41**	TYR	GLN	498	3.937															
	TYR	THR	500	2.866															
	TYR	ASN	501	2.831															
**42**	GLN	GLY	446	3.361															
	GLN	TYR	449	2.882															
	GLN	GLN	498	3.212															
**79**					THR					ILE					HIS				
**82**	MET	PHE	486	3.545	SER	PHE	486	3.602	+	ASN	PHE	486	3.584	+	THR	PHE	486	2.944	-
**83**	TYR	PHE	486	3.232	PHE	PHE	486	3.446	+	PHE	PHE	486	3.453	+					
	TYR	ASN	487	2.947															
**353**	LYS	GLY	496	2.899															
	LYS	ASN	501	3.762	HIS	ASN	501	3.741	+	HIS	ASN	501	3.737	+					
	LYS	GLY	502	2.847	HIS	GLY	502	2.853	-	HIS	GLY	502	2.849	-					
	LYS	TYR	505	3.339	HIS	TYR	505	3.370	+	HIS	TYR	505	3.367	+					
**354**	GLY														ARG	ASP	405	2.858	-
	GLY														ARG	GLY	502	3.483	-
	GLY																		
	GLY														ARG	TYR	505	3.400	-
**355**	ASP	THR	500	3.503															
**357**	ARG	THR	500	3.437															
